# Order recognition by Schubert polynomials generated by optical near-field statistics via nanometre-scale photochromism

**DOI:** 10.1038/s41598-022-21489-6

**Published:** 2022-11-08

**Authors:** Kazuharu Uchiyama, Sota Nakajima, Hirotsugu Suzui, Nicolas Chauvet, Hayato Saigo, Ryoichi Horisaki, Kingo Uchida, Makoto Naruse, Hirokazu Hori

**Affiliations:** 1 University of Yamanashi, 4-3-11 Takeda, Kofu, Yamanashi 400-8511 Japan; 2 Department of Information Physics and Computing, Graduate School of Information Science and Technology, The University of Tokyo, 7-3-1 Bunkyo-ku, Tokyo, 113-8656 Japan; 3 Nagahama Institute of Bio-Science and Technology, 1266 Tamura, Nagahama, Shiga 526-0829 Japan; 4 Ryukoku University, 1-5 Yokotani, Oe-cho, Seta, Otsu, Shiga 520-2194 Japan

**Keywords:** Mathematics and computing, Nanoscale devices, Optics and photonics, Adaptive optics

## Abstract

Irregular spatial distribution of photon transmission through a photochromic crystal photoisomerized by a local optical near-field excitation was previously reported, which manifested complex branching processes via the interplay of material deformation and near-field photon transfer therein. Furthermore, by combining such naturally constructed complex photon transmission with a simple photon detection protocol, Schubert polynomials, the foundation of versatile permutation operations in mathematics, have been generated. In this study, we demonstrated an order recognition algorithm inspired by Schubert calculus using optical near-field statistics via nanometre-scale photochromism. More specifically, by utilizing Schubert polynomials generated via optical near-field patterns, we showed that the order of slot machines with initially unknown reward probability was successfully recognized. We emphasized that, unlike conventional algorithms, the proposed principle does not estimate the reward probabilities but exploits the inversion relations contained in the Schubert polynomials. To quantitatively evaluate the impact of Schubert polynomials generated from an optical near-field pattern, order recognition performances were compared with uniformly distributed and spatially strongly skewed probability distributions, where the optical near-field pattern outperformed the others. We found that the number of singularities contained in Schubert polynomials and that of the given problem or considered environment exhibited a clear correspondence, indicating that superior order recognition is attained when the singularity of the given situations is presupposed. This study paves way for physical computing through the interplay of complex natural processes and mathematical insights gained by Schubert calculus.

## Introduction

Nanophotonics has been extensively studied not only for energy^1^, lighting^2^, or sensing^3^ applications but also in view of information physics and computing^4–8^ to benefit from light-matter interactions at the subwavelength scale. Optical near-field interactions play a crucial role in the field of nanoscale materials^9^. Indeed, nanoscale logic devices have been theoretically and experimentally demonstrated based on excitation transfer via optical near-field interactions^10^. Moreover, the examination of metamaterials for computing is actively discussed^6^. Furthermore, higher-order intelligent functionalities, such as solving satisfiability problems^11^ and decision-making problems^12^, are investigated using optical near-field interactions.

In contrast, extremely high precision nano-fabrication technologies are indispensable to realizing such nanophotonic devices^13^. However, to date, technical difficulties in terms of ultrafine individual control of the size and position of nanomaterials, such as quantum dots, are still difficult to resolve; this is one of the primary reasons for the development of self-assembly techniques^14,15^. Additionally, memory function is another critical factor in view of constructing computing functionality.

**Figure 1:**
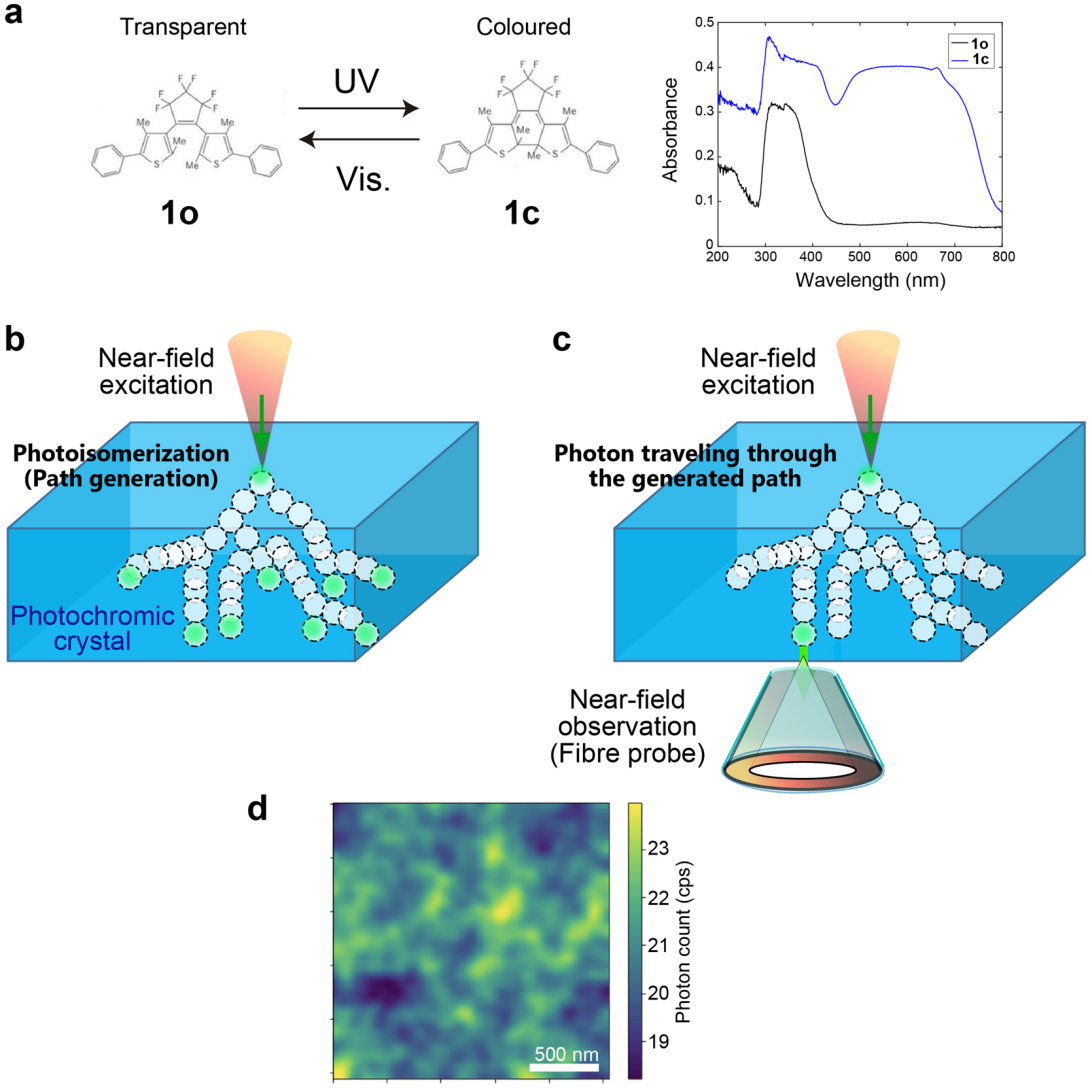
Nanoscale photochromism by near-field optics. (**a**) Transparent and coloured states of diarylethene and their absorption spectra. (**b**) Local near-field light excitation at the surface of the photochromic material to generate photoisomerization at the nanometre scale. Complex pathways are developed. (**c**) Once th pattern is formed, the local optical excitation travels through the generated path. (**d**) The output position of the photons was observed using scanning near-field optical microscopy. Adapted from Uchiyama et al.^18^, Sci. Rep. 10, 2710 (2020). Copyright 2020 Author(s), licensed under a Creative Commons Attribution 4.0 License.

Considering these issues, we focus our attention on photochromic materials^16^, which exhibit light-induced reversible transformations between transparent or colourless open-ring isomers (1,2-bis(2,4-dimethyl-5-phenyl-3-thienyl)-3,3,4,4,5,5-hexafluoro-1-cyclopentene; **1o**) and opaque or blue-coloured closed-ring isomers (**1c**) (Fig. 1a). Visible light irradiation induces the isomerization from **1c** to **1o**, whereas ultraviolet (UV) light demonstrates an opposite effect. Photoisomerization assumes the role of information memorization. As shown in Fig. 1a, the absorption spectra of colourless and blue-coloured opaque states of the crystalline surface were measured through the diffuse reflection method using an integrating sphere.

Furthermore, it should be emphasized that the formation of an autonomous pattern has been realized in forming a nanometre-scale ultrafine structure instead of pixel-wise addressing, as in conventional optical memory. To fully benefit from subwavelength-scale photonics, we utilized optical near-fields and locally induced photoisomerization of photochromic materials^17,18^. Indeed, in a previous study^17^, Nakagomi et al., demonstrated that a local optical near-field excitation on the surface of a photochromic crystal yields local photoisomerization on the scale of tens of nanometres. Initially, the entire material was in a coloured state (**1c**). Through an optical near-field excitation with a visible wavelength that was fixed at a spatial position on the crystal surface, the material was locally isomerized into a transparent **1o** state. The locally induced transparency guides the incoming optical near-field into a neighbouring area in the subwavelength regime. Consequently, the local photoisomerization of the transparent state causes a succeeding chain reaction of local transparency, including bifurcations, leading to the formation of a complex pattern of transmitted photons on the opposite surface of the crystal (Fig. 1b).

Once the nanoscale transparency paths are formed, an incoming near-field excitation is transferred to the opposite side and scattered by the probe tip located at a particular position (Fig. 1c). Figure 1d shows the experimentally observed photon count statistics in a 2 μm × 2 μm regime taken by a scanning near-field optical microscope (SNOM) with a spatial resolution of approximately 50 nm. We observed non-uniform distributions stemming from the complex structure formed in the photochromic materials^17^.

Although the photon count pattern appears complicated, internal photoisomerization is triggered by the local excitation from a single fixed position. Therefore, photon statistics are not random but comprise certain spatial correlations. The origin of the complexity stems from the interplay between local photoisomerization by an optical near-field and its subsequent anisotropic deformation of the molecular size, leading to anisotropic mechanical strain^17^. Such a structure is, metaphorically speaking, as if chaos provided complex profiles originating from simple common deterministic nonlinear dynamics. By exploiting these characteristics, Uchiyama et al.^18^ have successfully demonstrated the generation of Schubert polynomials using the photon count statistics combined with a simple photon detection protocol. Here, the experimentally observed optical near-field data was common for the previous studies^17,18^ and the present study.

However, Uchiyama et al.^18^ only described the production of Schubert polynomials; its mathematical properties, formulated as Schubert calculus, were not fully utilized. Furthermore, quantitative comparisons with other random numbers, such as uniformly distributed pseudorandom numbers, were not performed. Therefore, in the present study, we demonstrated order recognition using Schubert polynomials generated by the experimentally observed optical near-field pattern.

Here, the issue of order recognition concerns the following situation: there are *N* slot machines whose reward probabilities are given by *P*_*i*_ (*i* = 1, …, *N*). The task of order recognition is to realize the ranking of all *P*_*i*_ in an ascending or descending order. In this study, we proposed an order recognition strategy based on Schubert calculus. The primary advantage of the proposed strategy is that it never calculates the estimated reward probability $\hat{P}_{i}$ while evaluating the order; instead, the proposed method highlights the relative relation between two choices, which is determined by an *inverse* relationship in a Schubert polynomial^19–21^. Furthermore, we quantitatively analyzed Schubert polynomials and their order recognition capabilities depending on the property of the source of the randomness. We showed that the number of *singularities* contained in the generated Schubert polynomials correlated with the order recognition capability.

In a general context, the contributions of the present study are based on Schubert calculus and complex optical near-field photon to physics-based computing from the following perspectives: the first contribution is the capability of producing versatile Schubert polynomials that inherit the correlation induced in the photochromic material by local excitation. The number of possible Schubert polynomials reaches the order of Avogadro’s number (10^23^) or becomes extremely diverse when *N* reaches 24. Nevertheless, Schubert polynomials generated via the proposed physics-based protocol^18^ carry the spatial correlations formed in the subwavelength-scale material system.

Second is the newly designed ordering algorithm based on Schubert calculus, a solid theoretical foundation of an ordered structure^19–21^. As mentioned earlier, the proposed algorithm consists of repeating the revision of orders between two choices, which mathematically corresponds to the so-called divided difference operation in Schubert calculus^19–21^. The transformation of the divided difference operator into an order recognition strategy is the original contribution of the present study.

Third, through the analysis of the comparison among the spatially uniform, spatially highly skewed, and optical near-field patterns, we found that ordering performs well when the singularity in the given problem and that in the source patterns meet each other. In other words, excellent performance results are observed when the problem under study and the solver are in an adjoint relationship. Although much deeper insights are needed from future studies, these contributions of the present study shed light on the utilization of naturally constructed complex information inherent at the subwavelength scale for intelligent functions. Based on this fundamental nature, the proposed strategy may be extended to other applications in the future, not just for order recognition of slot machines or bandit problems examined in the present study.

The remainder of the paper is organized as follows: first, we review the optical near-field statistics observed using a double-probe optical near-field microscope^17^. We then review the generation of Schubert polynomials based on near-field observation coupled with a simple photon detection protocol^18^. The order recognition strategy based on Schubert calculus is then introduced, including the demonstrations using the experimentally observed optical near-field pattern. We also quantitatively compare order recognition abilities with random number distributions, such as uniformly distributed random numbers.

## Results

### Preparation (1): observation of a complex optical near-field transmission pattern on the photochromic crystal

We reviewed the results of previous studies in the present study. First, we summarized the complex photon path formation in photochromic materials^17^. As mentioned above, the material was a photochromic single crystal made of diarylethene, with the molecular structure shown in Fig. 1a^22,23^. Upon UV irradiation, the molecule was isomerized from a transparent isomer (**1o**) into a blue-coloured isomer (**1c**) while maintaining the crystal structure^24,25^. Upon visible light irradiation, the material was converted back into a colourless or transparent isomer. The black and blue curves in Fig. 1a represent the absorption spectra of isomers **1o** and **1c**, respectively. The molecular lengths of the two carbon atoms at 4- and 4’’- positions in the phenyl rings of **1c** and **1o** were 1.39 nm and 1.41 nm, respectively, and the thicknesses of the molecules **1c** and **1o** were 0.39 nm and 0.49 nm, respectively^25^, indicating that structural deformation accompanies photoisomerization.

The local optical excitation and its near-field optical transmission characterization were conducted using a double-probe scanning near-field optical microscope (Unisoku, USM-1300S), which has a metallic probe on one side and an apertured fibre-optic probe on the other side (Fig. 1c). With the sharpened gold-coated probe tip located near the surface of the diarylethene crystal with a thickness of 100 μm, local optical excitation was induced using light with the wavelength of 532 nm^17,18^. Here, we utilized the local electric-field enhancement at the tip of the sharpened metallic probe^17,26^. The near-field transmission was measured using an optical fibre probe with a spatial resolution of approximately 50 nm, which was connected to a single photon counting apparatus. The two-dimensional scanning area was 2 μm × 2 μm with a resolution of 256 pixels × 256 pixels.

Figure 1d shows the observed near-field photon count distribution obtained after adopting a two-dimensional Gaussian filter with a standard deviation of 6 pixels (approximately 50 nm), corresponding to the resolution limit of the system. We observed complex subwavelength structures with a representative scale of 100–200 nm. In particular, the local excitation by the metallic tip did not uniformly propagate through the material. The generation of such a complex pattern was attributed to the balance between the mechanical deformation of the photochromic material and photoisomerization^17,23^. This suggests that local photoisomerization induces anisotropic deformation of the molecular size, leading to anisotropic mechanical strain^27,28^, which causes subsequent photoisomerization in a non-uniform manner in the surrounding material.

In other words, a chain of spontaneous symmetry breakings was generated during local photoisomerization involving branching and selection; the near-field–excited diarylethene was locally modified to be transparent. The developed transparent area was accompanied by an anisotropic mechanical strain in the adjacent area, leading to a suppressed or enhanced photoisomerization of the subsequent photons. Therefore, an anisotropic strain field from the adjacent locally photoisomerized areas provided an anisotropic spatial distribution of the quantum yield of photoisomerization within the crystal^17^. Such chains of photoisomerization and strain-field formation led to nanometre-scale transparent paths, resulting in the generation of versatile patterns observed in Fig. 1d. After generating a transparent path, the two-dimensional near-field observation was conducted with the input light from the metallic tip at a single photon level, with photon energies lower than the limit to induce further photoisomerization. Diarylethene used in this study does not exhibit thermal isomerization from the closed- to open-ring isomer or vice versa^29^; therefore, the constructed nanometre-scale pattern was preserved until complete resetting via strong UV or visible light irradiation was observed.

As reported by Nakagomi et al.^17^, the metal probe was illuminated using 532-nm laser light with an intensity of 1 μm/cm^2^. The number of photons detected by the optical near-field fibre probe was in the range of 10 counts per second (cps) from approximately 100-nm order spatial area. Although the principal contribution of the present study was constructing an order recognition strategy, we briefly discussed the photon utilization efficiency of the experiment described in the study by Nakagomi et al.^17^, which was not discussed in previous studies. Future studies with a detailed experimental discussion are warranted.

For simplicity, assuming that the local excitation occupies an approximately 100-nm square area and the tip-enhancement factor by the gold-coated probe is 100, the number of photons that are subjected to the nanometre-scale transparent paths formed in the photochromic crystal per second is approximately 30,000 or the input photon rate is 30,000 cps. Furthermore, assuming that the input photons can travel in the 5-μm square area of the sample, the number of photons observed from a 100-nm square area is approximately 12 cps. Interestingly, this number is comparable to the experimentally observed photon counts by SNOM, meaning that, the intermediate loss is negligible. However, if the tip enhancement by the gold probe is estimated to be 1000, the output photon should be 120 cps, meaning that the loss is approximately 90%. As mentioned above, detailed characterization is warranted in future studies.

### Preparation (2): generation of the Schubert matrix

A single photon travels through the generated complex transparent paths and is finally transmitted from a particular position to the opposite side. The spatial position of the photon detection differs from photon to photon, and Fig. 1d represents its statistics. Uchiyama et al.^18^ reported that, although the spatial positions of the photon observations were versatile, it was constrained by the transparent paths formed in the diarylethene crystal, which were originally specified by a fixed local excitation from the metallic probe. To transform the spatial near-field photon statistics into versatile information in a coherent manner, Uchiyama et al.^18^ incorporated the following simple photon detection protocol to generate Schubert polynomials, which were equivalent to what they called “*Schubert matrices*”, as introduced in the present study.

#### Schubert matrix

In a Schubert matrix, there is only a single element of 1 for all rows and columns, while all the other elements are 0. In other words, a Schubert matrix represents a permutation. the Schubert matrix has a one-to-one correspondence with the Schubert polynomial^19^. See the “Methods” section for details of the definition of Schubert polynomials. Schubert matrices of the size *N* × *N* were utilized for recognizing the order of *N* distinct slot machines. As given by Eqs. (3)–(5), a Schubert polynomial corresponding to an *N* × *N* Schubert matrix is a polynomial composed of the term $x_{1} ,x_{2} , \ldots ,x_{N - 1}$.

#### Basic notions: diagram, inversion, Young tableau, and singularity

**Figure 2:**
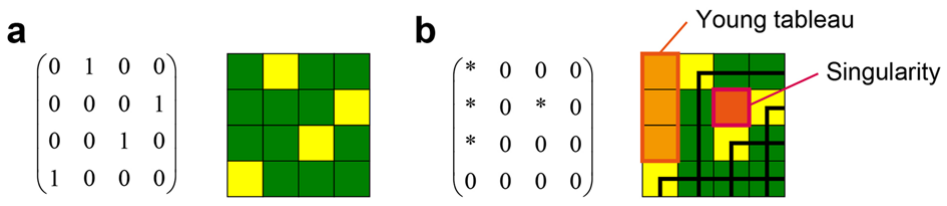
Schematic of an example of the Schubert matrix. (**a**) The permutation of [2431] is represented by a matrix *S* in Eq. (2). (**b**) The *diagram* of the matrix *S* is illustrated. The upper-left block consisting of $*$ s (indicated by orange squares) is called the *Young tableau*, whereas isolated $*$ blocks are called *singularities*.

Herein, we introduce a few basic notions to characterize Schubert matrices: *diagram*, *inversion number*, and *singularity*. An example of a Schubert matrix is a 4 × 4 matrix given by

1 $$S = \left( {\begin{array}{*{20}c} 0 & 1 & 0 & 0 \\ 0 & 0 & 0 & 1 \\ 0 & 0 & 1 & 0 \\ 1 & 0 & 0 & 0 \\ \end{array} } \right),$$

which is illustrated in Fig. 2a with yellow and green elements denoting 1 s and 0 s, respectively. Herein, we marked the elements that are located on the *right* and *lower* sides of the yellow elements, which are marked by thick black lines in Fig. 2b. Therein, the non-marked elements are called the *diagram* of the Schubert matrix, which in this case is given by

2 $$\left( {\begin{array}{*{20}c} * & 0 & 0 & 0 \\ * & 0 & * & 0 \\ * & 0 & 0 & 0 \\ 0 & 0 & 0 & 0 \\ \end{array} } \right).$$

The number of $*$ s in the diagram is called the *inversion number*, which is 4 in the case of the present example. An array of $*$ s located at the upper-left corner of the matrix is called *Young tableau*^30^. Furthermore, in the diagram, let us connect $*$ s into blocks if they are connected by any of their four neighbours: up, down, left, or right. We then count the number of blocks of connected $*$ s, which is 2 in the case of Eq. (2). Here, the number of *singularities* is defined by the number of such blocks without counting the Young tableau. In the case of Eq. (2), there are two blocks of $*$, with one of them being the Young tableau. Therefore, the singularity is 1.

#### How to generate a Schubert matrix

**Figure 3:**
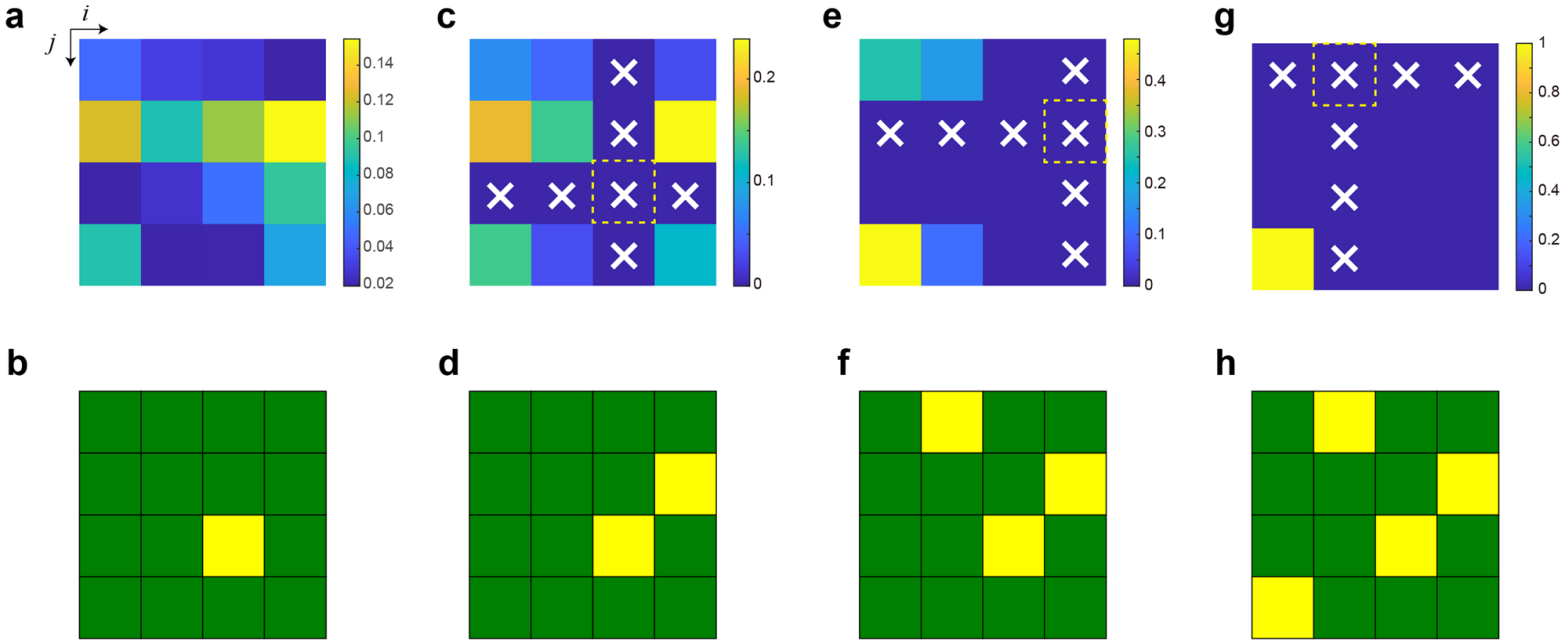
Generation of Schubert polynomials based on optical near-field observation. (**a**) The detection area is divided into *N* × *N* elements. Here *N* = 4. The photon detection probability is spatially irregular. (**b**) When a photon is detected at an element (*i*, *j*), the (*i*, *j*)-element of the Schubert matrix is given by 1. Here, (*i*, *j*) = (3,3). (**c**) The sensitivities of all the elements in the third row and column are zero. The probabilities are renormalized. (**d**) The next element of the Schubert matrix is determined. (**e**–**h**) The process described in (**c**) and (**d**) is repeated. (**h**) is the finally obtained Schubert matrix in this example. Adapted from Uchiyama et al.^18^, Sci. Rep. 10, 2710 (2020). Copyright 2020 Author(s), licensed under a Creative Commons Attribution 4.0 License.

The generation of a Schubert matrix *S* is described in the study by Uchiyama et al.^18^. Here, we introduced the procedure along with a case of generating a Schubert matrix with a size of 4 × 4. As previously illustrated, a single photon was observed at a spatial position with the corresponding probability distribution (Fig. 3a). The details of the conversion from the experimentally observed photon statistics to selection probability are described in the previous study^18^.

1. The probability distribution specified by the near-field observation was rescaled or coarse-grained into an *N* × *N* grid. The probability at each point of the grid is denoted by *P*_*i*,*j*_ (*i* = 1,…, *N*, *j* = 1,…, *N*) hereafter, where *N* is a natural number. In the example, *N* is equal to 4 (Fig. 3a). In the physical system shown in Fig. 1c, when sending a single photon, we observed the photon at one of the grid points. In this study, by using uniformly distributed pseudorandom numbers generated in a computer and pondered by the measured probabilities, we emulated the output photon observations.
2. We counted the number of photons detected at each grid element. Let the photon count at the grid point (*i*, *j*) be denoted by *C*_*i*,*j*_ (*i* = 1,…, *N*, *j* = 1,…, *N*). When the photon count at a certain position *C*_*m*,*n*_ reaches the threshold value denoted by *P*_T_, we determined that the (*m*, *n*)-element of the Schubert matrix is given by 1; *S*_*m*,*n*_ = 1. In the example shown in Fig. 3b, *S*_3,3_ = 1.
3. Accordingly, the photon detection probability distribution *P*_*i*,*j*_ was updated for the rest of the loop. First, photon detection at the *m*-th row and the *n*-th column was configured to be zero, which are denoted by x marks in Fig. 3c, i.e., *P*_3,*j*_ = 0 (*j* = 1,…, 4) and *P*_*i*,3_ = 0 (*i* = 1,…, 4). This reconfiguration was based on the definition of the Schubert matrix. Second, the probability distribution *P*_*i*,*j*_ was renormalized such that $\sum\nolimits_{i,j} {P_{i,j} } = 1$ was maintained.
4. Similar to Step (2) described above, when *C*_*m’*,*n’*_ reaches *P*_T_, we determined the (*m’*, *n’*)-element of the Schubert matrix to be 1, i.e., *S*_*m’*,*n’*_ = 1. In the example shown in Fig. 3d, *S*_2,4_ = 1.
5. We repeat steps (2) and (3) for two times (Fig. 3e–h); *S*_1,2_ = 1 is determined as shown in Fig. 3f, followed by *S*_4,1_ = 1 in Fig. 3h.

The final determination of *S*_1,2_ = 1 is immediately given after the previous step because only a single grid point has a non-zero photon observation probability. The resultant Schubert polynomial is $x_{1}^{2} x_{2} x_{3} + x_{1} x_{2}^{2} x_{3}$; see the “Methods” section for information regarding Schubert polynomials^19^.

The total number of different Schubert matrices of size *N* × *N* is given by *N*!, meaning that the factorial order increase with the size of the observation elements. With *N* = 32, the total number is more than 10^35^. The ability to generate such an extremely diverse pattern from the same substrate via optical near-field processes was the primary topic investigated by Uchiyama et al.^18^.

The photon number threshold *P*_T_ played an important role. A smaller *P*_T_ value leads to diverse choices of 1 s in the generated Schubert matrix. Conversely, a larger *P*_T_ value indicates that the element with the maximum photon detection probability most likely yields the value of 1 in the resulting Schubert matrix. In other words, the resultant Schubert matrices are more weakly and more strongly correlate with the source optical near-field pattern with smaller and larger *P*_T_ values, respectively^18^. These are called *soft* and *hard* correspondences between the near-field patterns and Schubert matrices, as described previously by Uchiyama et al.^18^.

### Order recognition

In previous sections, we reviewed the background and fundamentals needed for the generation of Schubert matrices based on near-field photon statistics from a photochromic material. The primary focus of this study is to utilize these Schubert matrices for information processing; specifically, our main objective is to recognize the order or ranking of slot machines with an initially unknown reward probability.

#### Order recognition by the revision of the name and value

**Figure 4:**
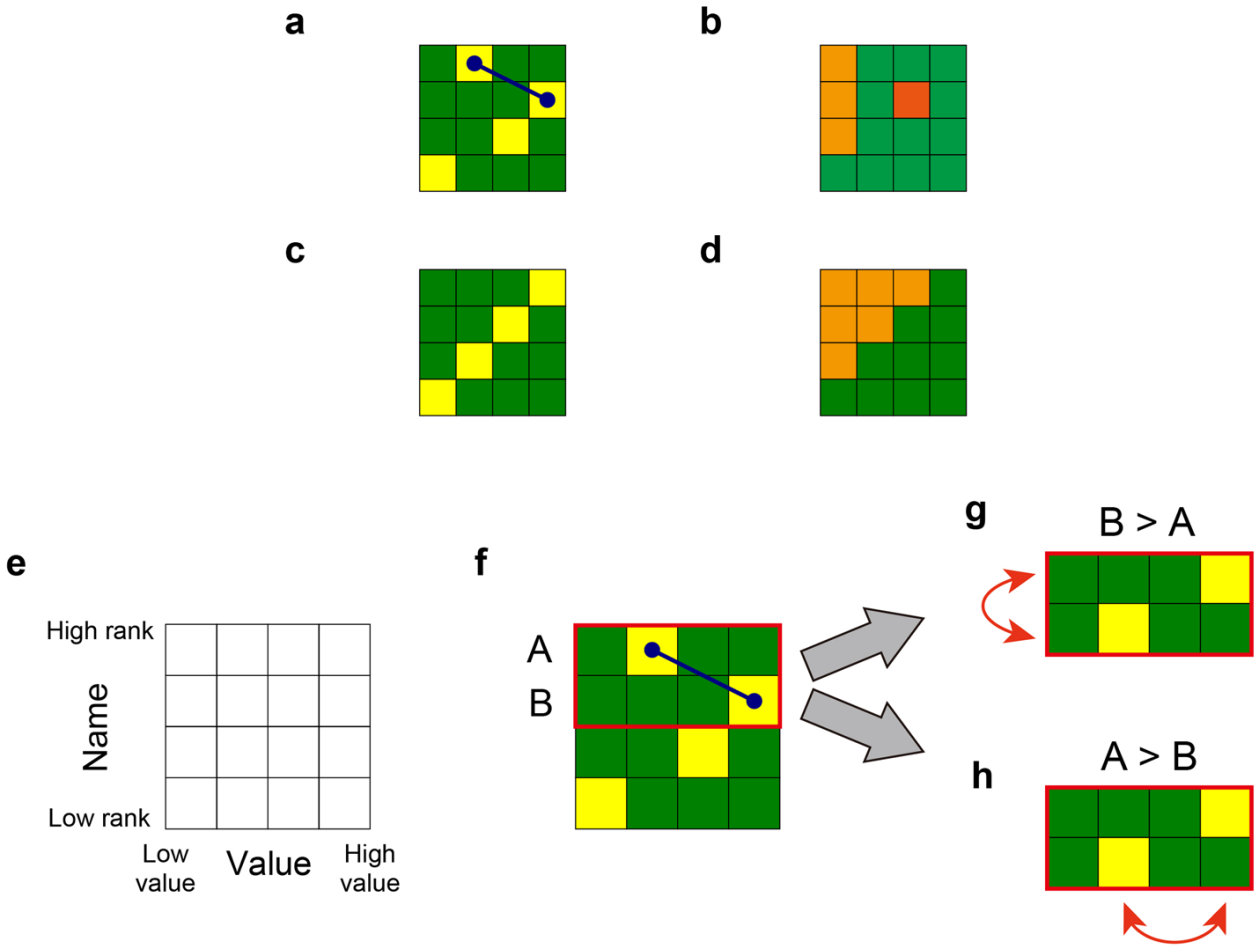
Order recognition principle based on Schubert polynomials. (**a**) Inversion relation (marked by a blue arrow) inherent in the Schubert matrix. (**b**) Young tableau (orange colour) and a singularity (red colour). (**c**) Illustration of an anti-diagonal matrix and (**d**) its diagram consists of a Young tableau with the maximum inversion number. (**e**) In the proposed ordering strategy, a matrix is characterized by the *name* and *value* axes, which correspond to the vertical and horizontal directions, respectively. In the name axis, the upper row indicates a high rank and in the value axis, the right column indicates a high value. (**f**) Our decision of machine selection is suggested by the Schubert matrix. We paid attention to the inversion relation of the given matrix, which is the first and the second row in (**f**). Let us assume that the names of the first and second rows be machines A and B, respectively. The inversion relation suggests a contrary situation between the name and value axes. (**g**) If the reward dispensed by machine B is greater than that by machine A, the association of the name axis is inaccurate; hence, the allocation of the names is swapped. (**h**) If the reward by machine A is larger than that by machine B, the association of the value axis is inaccurate; therefore, the allocation of the values is swapped.

The key is to exploit the inversion relation presented in a Schubert matrix; here, we introduce a 4 × 4 matrix, which is schematically illustrated in Fig. 4a. If the column number of the element with the value of 1 in the *i*-th row is smaller than that in the (*i* + 1)-th row, we define that these two elements are in a relation of *inversion*. Furthermore, this inversion is related to the concept of *singularity*, as described above. The diagram of the Schubert matrix (Fig. 4a) is shown in Fig. 4b.

Conversely, when the Schubert matrix is represented by an anti-diagonal matrix (Fig. 4c), there are no inverse relations. The diagram of an anti-diagonal matrix is shown in Fig. 4d, where the upper triangular elements and the Young tableau only contribute to forming the inversion number. The inversion number is maximized, and there is no singularity. The Schubert polynomial is represented by a single term $x_{1}^{3} x_{2}^{2} x_{3}^{{}}$.

As described above, inversion relations and singularities indicate characteristic properties. The idea of the proposed ordering strategy based on Schubert calculus begins with associating information to the vertical (row) and the horizontal (column) axis of Schubert matrices.

Let us assume that there are four slot machines (*M*_1_, *M*_2_, *M*_3_, *M*_4_) and their reward probabilities are given by (*P*_1_, *P*_2_, *P*_3_, *P*_4_) = (0.4, 0.2, 0.8, 0.6), which are unknown to us. In this case, the *ground truth* order of the slot machines’ reward probability is *M*_3_, *M*_4_, *M*_1_, and *M*_2_ in descending order. We want to recognize this ground truth order by playing the slot machines.

The first critical point of the proposed order recognition strategy is to associate the vertical (row) and horizontal (column) axes of a matrix with the *name* and *value*, respectively. Specifically, the names of the slot machines are bound with the rows; the upper rows indicate a higher-ranked machine, while the lower rows indicate a lower-ranked machine. Meanwhile, the columns correspond to the reward probabilities, with the right column being the high reward probability. These associations are illustrated in Fig. 4e. In the pseudo-code shown below, these associations are represented by line 5.

A Schubert matrix *proposes* the relation between the name and value by the elements of 1. Assuming that the elements in the matrix along the name axis are perfectly arranged in the order of the ground truth, and the elements along the value axis are arranged in the order of the ground truth reward probabilities in the ascending order, then, the matrix results in an anti-diagonal matrix, as shown in Fig. 4c. Here, there are no inverse relations and no singularities, suggesting that, the matrix for the perfect order recognition is an anti-diagonal matrix. Therefore, transforming the initially unsure order regarding both the name and value axes to adapt the correlated Schubert matrices toward an anti-diagonal matrix corresponds to accomplishing the correct order recognition. The question now is how to realize such transformations.

We considered that Schubert matrices *propose* which machines should be checked to determine the correct order. In this study, two machines are selected for a single-slot machine play. This is similar to the multi-armed bandit (MAB) problem with multiple plays^31^, although the aim of solving MAB problems and that in the present study are fundamentally different. The purpose of solving MAB problems is usually to minimize regret, whereas the objective of the present study is to recognize the order.

The machine selection was determined by the inverse relation in the Schubert matrix that provided the deviation from the anti-diagonal matrix. In the example shown in Fig. 4a, there is one inverse relation indicated by a solid blue arrow.

We now focus our attention on the inverse relation. In the example shown in Fig. 4a, the inversion denotes the first and the second rows. Let us assume that the names of the first and second rows are machine A and machine B, respectively. Based on the definition of the vertical and horizontal axes of the matrix shown in Fig. 4e, the relation between machines A and B is *contradictory* (Fig. 4f). According to the name axis (vertical), machine A was considered a higher reward machine. However, the value axis (horizontal) indicated that the value of machine B was greater than that of machine A, suggesting that the relationship is contradictory.

To resolve such a contradiction, we selected these two machines and checked the betting results.

[CASE 1]: When the reward dispensed by machine A is greater than that of machine B:
  The order of the name axis is correct; machine A is above machine B. In contrast, the value axis is *wrong*; the value of machine A should be on the right-hand side of machine B. Therefore, we *swapped* the allocation of the values, leading to a situation depicted in Fig. 4g.
[CASE 2]: When the reward dispensed by machine B is greater than that of machine A:
  The order of the value axis is correct; the value of machine B is on the right-hand side of machine A. Conversely, the name axis is *wrong*; machine B should be above machine A. Therefore, we *swapped* the allocation of the names, leading to a situation depicted in Fig. 4h.

The resulting arrangement is the same as shown in Fig. 4g and h for both cases; however, the reconfiguration of the axes is contrasting.

After finishing the reconfiguration using the current Schubert matrix, we continued the process by using the next Schubert matrix, which is shown in line 5 in the pseudo-code below (*S*← Schubert matrix). Furthermore, the row and column of the new Schubert matrix were transformed by the name and value axes to date, which is represented by line 5 in the pseudo-code (*S*← *S*[name,value]).

The above-described principle is summarized as a pseudo-code by the following algorithm: variable *t* is incremented after a single loop is completed, as shown by line 18 in the pseudo-code. A single Schubert matrix is used in a single loop given by line 5.
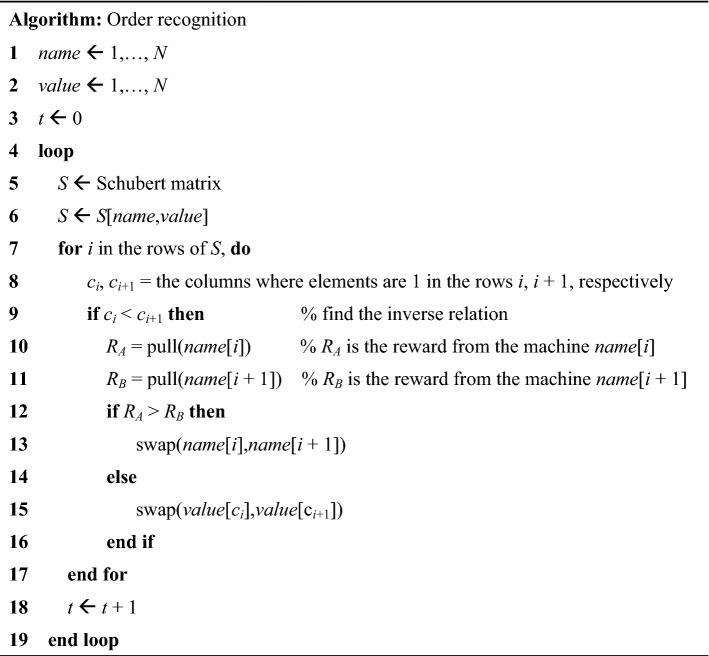


### Demonstration

**Figure 5:**
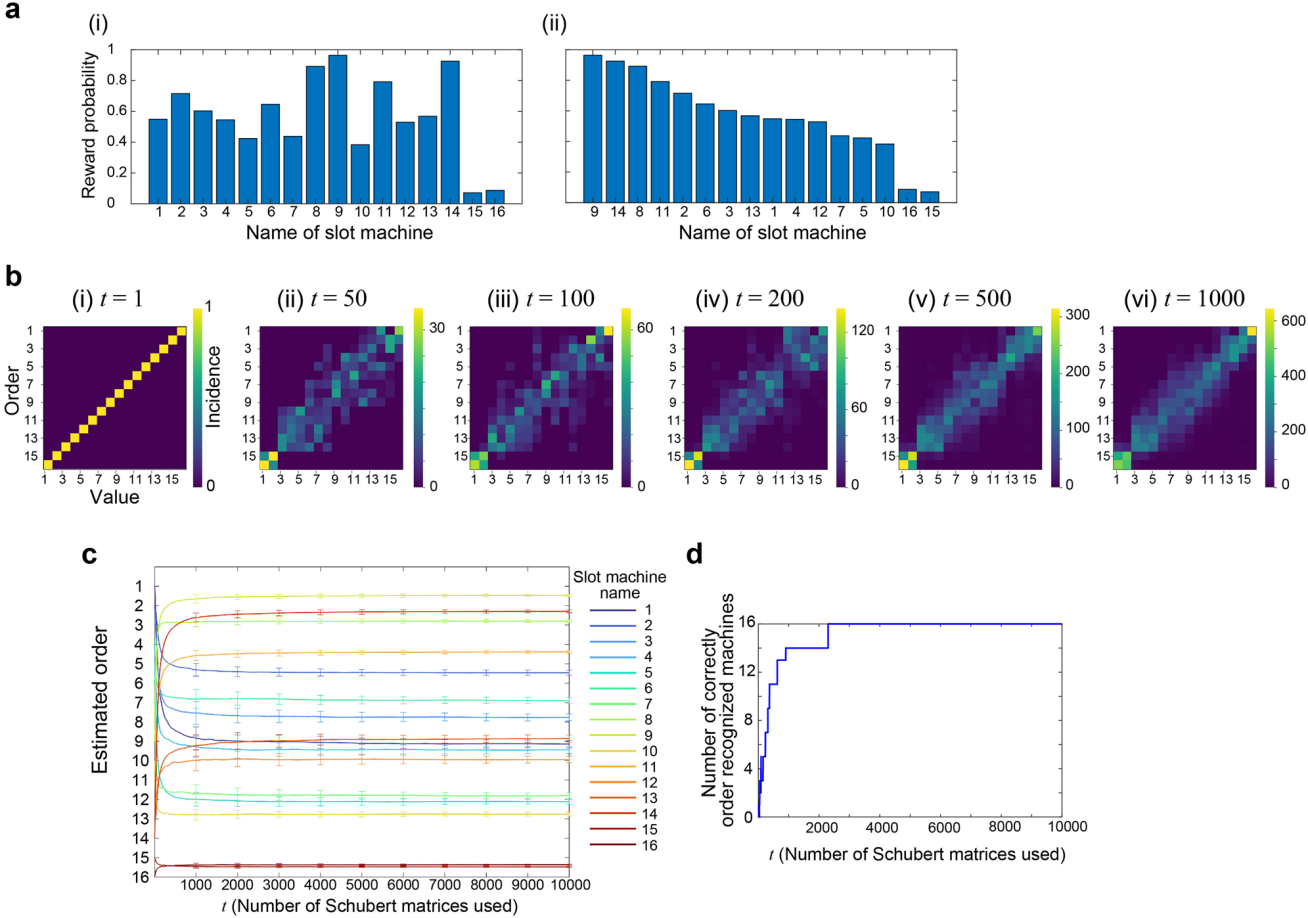
Demonstration of order recognition in 16 slot machine environments. (**a**) (i) Reward probability arrangement and (ii) its sorted representation. (**b**) The accumulated incidence pattern of the ranking-and-value matrix after the loop of (i) 1, (ii) 50, (iii) 100, (iv) 200, (v) 500, and (vi) 1000. (**c**) The time evolution of the estimated ranking of each slot machine. (**d**) The number of correctly ordered machines over time.

We demonstrated the order recognition with 16 slot machines (*M*_1_–*M*_16_) with the ground truth reward probability setting of (*P*_1_–*P*_16_), as shown by the bar graph in Fig. 5a(i). The true order of the reward probability in the descending order is given by *M*_9_, *M*_14_, *M*_8_, *M*_11_, *M*_2_, *M*_6_, *M*_3_, *M*_13_, *M*_1_, *M*_4_, *M*_12_, *M*_7_, *M*_5_, *M*_10_, *M*_16_, and *M*_15_, as shown in Fig. 5a(ii).

Initially, the association of the name axis starts with the linear order corresponding to the index of the machines, i.e., *M*_1_–*M*_16_. We used a total of 10,000 Schubert matrices consecutively. This whole procedure was repeated for *R* = 100 trials. As the reward dispensed by each machine was probabilistic, a varying win–lose event occurred among *R*-times trials.

The images in Fig. 5b demonstrate how order recognition evolves as time elapses. Specifically, the horizontal axis indicates the number of Schubert matrices used, which is equal to the elapsed number of loops and is denoted by *t* using the notations shown in lines 3 and 18 of the Algorithm.

Here, the order of machine *M*_*i*_ at time *t* was evaluated by calculating how often the machine *M*_*i*_ is associated with the row number of the Schubert matrices. Meanwhile, we computed how the value of *M*_*i*_ was evaluated at time *t* by analyzing how its value was ranked in the horizontal direction.

The two-dimensional images in Fig. 5b show how the order recognition evolves by counting the accumulated incidences of the matrix (ranking and value), referred to as the *ranking-and-value matrix* hereafter, at *t* = 1, 50, 100, 200, 500, and 1000. If the order recognition is completed to perfection, the top-ranked machine owns the highest value, whereas the worst-ranked machine has the minimum value. Therefore, the ranking-and-value matrix exhibits higher incidences along the anti-diagonal elements.

As time elapses, the high incidence elements were gradually localized along the anti-diagonal axis, indicating successful order recognition. Immediately after the first round of the loop (*t* = 1), the ranking-and-value matrix was in an anti-diagonal arrangement (Fig. 5b(i)). This is because there was only a single incidence regarding both ranking and value axes at *t* = 1. As shown in Fig. 5b(ii), the ranking-and-value matrix shows a diverse incidence pattern at *t* = 50.

Figure 5c visualizes the results in another manner, where the vertical axis represents the estimated order, while the horizontal axis shows the number of Schubert matrices used or the time dimension. Precisely, the estimated order of machine *M*_*i*_ at time *t* was calculated by the average of the estimated ranking of *M*_*i*_ by the time *t*. Furthermore, such an estimated order is averaged over 100 different trials. The 16 curves therein, shown with different colours, represent the time evolution of how machine *M*_*i*_ was ranked. We observed that the estimated order was drastically reconfigured during the first approximately 500 steps. When the time step was around 2000, the order recognition was accomplished.

Furthermore, Fig. 5d summarizes the number of correct order-recognized machines as a function of time, which equals 16 when the perfect order recognition is achieved. Indeed, after *t* = 2298, the estimated order agreed with the ground truth ranking. We also observed that the order of most machines (14 out of 16) was recognized at *t* = 626. Therefore, the order recognition was almost completed at *t* = 626, although the perfect recognition was accomplished at *t* = 2298.

The error bars of Fig. 5c indicate the standard deviation of the estimated order; when the ground truth reward probability difference between the slot machines was minute, the error bars of associated machines overlapped with each other. We observed such a situation regarding *M*_15_ and *M*_16_, where the reward probability difference was only approximately 0.02.

However, we can automatically grasp such uncertainty by the ranking-and-value matrix. Indeed, we observed a high incidence square area or a block matrix in the lower-left corner of the matrix shown in Fig. 5b(iv), meaning that the ranking of the 15th and 16th machines are comparable. This is a remarkable attribute of the proposed strategy in which the strong correlations inherent in the problem under study are automatically detected.

Furthermore, as mentioned above, the proposed order recognition strategy relies only on the two choices specified by Schubert matrices and their resulting outputs. As opposed to the former order recognition principles^32^, the present study did not involve the notions of expected probabilities and confidence intervals.

## Discussion

### Comparison

**Figure 6:**
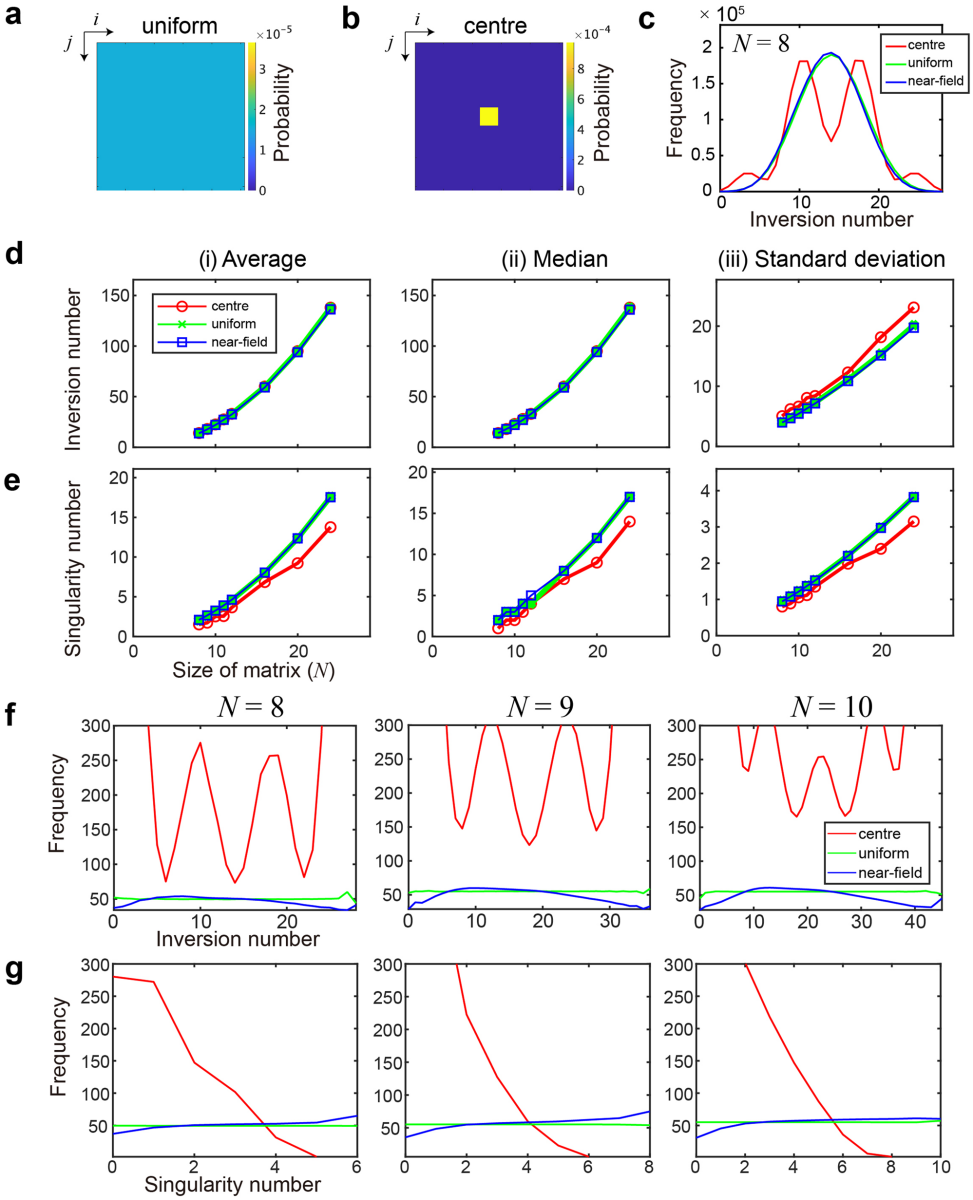
Characterization of Schubert polynomials generated by the near-field pattern compared with the (**a**) uniformly distributed and (**b**) spatially strongly skewed (centred) patterns. (**c**) Inversion number distribution when *N* = 8 by the near-field, uniform, and centre patterns. (**d**, **e**) Basic statistical analysis of the (**d**) inversion number and (**e**) singularity number as a function of the size of the matrix (*N*). (i) Average, (ii) median, and (iii) standard deviations are examined and no evident differences among the sources are observed. (**f**, **g**) When the incidence is normalized by the number of unique matrices, evident source dependencies are observed in both the (**f**) inversion number and (**g**) singularity number.

Here, we discuss the differences in Schubert matrices and their order recognition performances among various random number generators, especially, in a spatially uniform probability distribution called “*uniform*” (Fig. 6a) and a spatially strongly skewed probability pattern called “*centre*” (Fig. 6b). The latter has a more significant probability in the square area located at the centre. The detailed setting of the “centre” is described in the “Methods” section. The near-field photon observation pattern (Fig. 1d) is simply referred to as “*near-field*” hereafter.

The motivation behind the comparison among the near-field, uniform, and centre patterns is described as follows: the spatial inhomogeneity of the optical near-field observed from the locally excited photochromic crystal is one of the most significant characteristics, as shown in Fig. 1d, which was examined in detail in a previous study^18^. A contrasting counterpart is a spatially completely uniform distribution, from which we examined the difference between the uniform and near-field patterns.

The near-field pattern was *not* too spatially skewed, and a high-incident photon count area was spatially distributed. Based on these observations, we examined the case where the photon counts were strongly concentrated in a particular spatial area. By comparing such spatially extremely skewed cases and near-field patterns, we could examine the dependencies on spatial skewness. The centre pattern is an example of such a spatially skewed distribution. The size of the high-incident area and its position were also considered. We primarily focused on the centre pattern as a representative case in the present study.

#### Schubert matrix statistics

We generated Schubert matrices based on the near-field, uniform, and centre patterns with a *P*_T_ value of 1. Figure 6c shows the statistics of the inversion number when *N* is equal to 8. Herein, we generated 2 × 10^6^ Schubert matrices for each of the near-field, uniform, and centre patterns, for which inversion number statistics were depicted by the blue, green, and red curves in Fig. 6c, respectively. Both the near-field and uniform patterns exhibited symmetric incidences with a peak value having an inversion number of 14. The centre pattern showed a local minimum incidence when the inversion number was 14 and exhibited fluctuations as a function of the inversion number. The fluctuation is discussed later.

Although the centre pattern deviated from the near-field and uniform patterns, the average, median, and standard deviation exhibited almost the same value, as demonstrated in Fig. 6d, when *N* is equal to 8. Therefore, there are no evident differences from the viewpoint of such statistical perspectives.

Furthermore, we examined the statistics of Schubert matrices with large sizes of up to *N* = 24. The square, x, and circular marks in Fig. 6d(i) represent the average value of the inversion number of the generated Schubert matrices from the near-field, uniform, and centre patterns, respectively, as a function of the size of the matrix (*N*). The detailed setting of the generation of a Schubert matrix is described in the “Methods” section. Figures 6d(ii) and 6d(iii) summarize the median and standard deviation of the inversion number. To summarize, we did *not* observe significant source-signal dependencies in these basic statistics for the inversion number from Fig. 6d.

Similarly, we examined the average (Fig. 6e(i)), median (Fig. 6e(ii)), and standard deviation (Fig. 6e(iii)) of the singularity of the generated Schubert matrices from the near-field, uniform, and centre patterns, respectively. There were no explicit source-signal dependencies for singularity.

In contrast, evident source-signal dependencies were observed when the *uniqueness* of Schubert matrices was considered. The blue, green, and red curves in Fig. 6f(i) represent the frequency of Schubert matrices divided by the unique number of Schubert matrices as a function of their inversion number in the near-field, uniform, and centre patterns, respectively, when *N* was equal to 8. However, the uniform pattern yielded an almost identical frequency, regardless of the inversion number; the near-field pattern showed a peak frequency around the inversion number of 10. Similar trends were observed when *N* was equal to 9 and 10, as demonstrated in Fig. 6f(ii) and f(iii), respectively.

Similarly, the frequency of Schubert matrices divided by the unique number of Schubert matrices exhibited a similar tendency with respect to the number of singularities, as shown in Fig. 6g(i) (*N* = 8), Fig. 6g(ii) (*N* = 9), and Fig. 6g(iii) (*N* = 10). Although the uniform pattern displayed identical values for all singularities, the near-field pattern provided a more significant frequency as the singularity increased. The statistics were highly similar among these three source patterns. Therefore, the observation in Fig. 6g suggests that versatile Schubert matrices are generated through small singularities via the near-field pattern. Regarding the centre pattern, the generated Schubert matrices were mostly similar to each other when the number of singularities was small.

The near-field pattern was based on the experimentally observed photon statistics provided by Uchiyama et al.^18^, as demonstrated in Fig. 1d. The original data were obtained with the size of 256 × 256 pixels, which was rescaled to form *N* × *N* blocks when the size of the Schubert matrix was specified by *N* × *N*. When the original size of 256 pixels was not indivisible by *N*, the photon counts on the boundary were divided proportionally.

The underlying mechanism of the oscillatory behaviour of the inversion number generated in the centre pattern observed in Fig. 6c and f is not understood yet. However, we considered that the following properties may be involved: The centre pattern induces a high probability of generating non-zero elements in the centre area. Therefore, based on the construction protocol of the Schubert matrix, it is highly unlikely to have non-zero elements in the centre area’s north, south, west, and east sides. Hence, the resultant generated Schubert matrix inherits such a spatially highly skewed nature of the centre pattern.

#### Comparison of order recognition performances

We examined the order recognition performances depending on the source patterns regarding eight slot machine (*M*_1_–*M*_8_) environments. While the demonstrations showed so far suppose a probabilistic reward environment, here we assume the deterministic, different-valued rewards among (0.2, 0.3, 0.4, 0.5, 0.6, 0.7, 0.8, and 0.9). The correspondence between *M*_1_ to *M*_8_ and the rewards are randomly configured; we evaluated *K* = 8! = 40,320 types of unique correspondences between the machines and the rewards.

**Figure 7:**
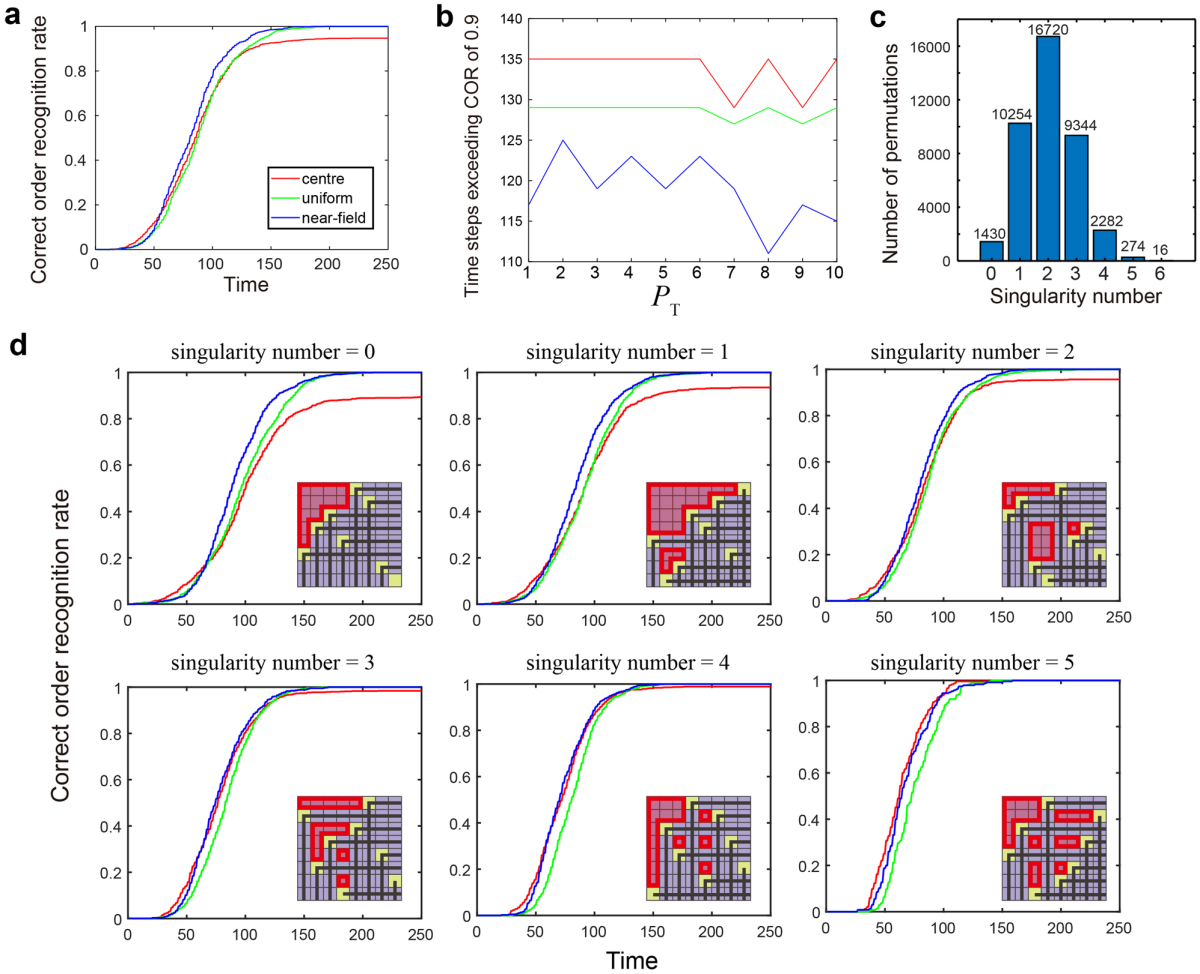
Order recognition performance evaluations with different source randomness using eight slot machine environments. (**a**) Time evolution of the COR rate for the near-field, uniform, and centre patterns. (**b**) The time steps exceed the COR value of 0.9 as a function of the photon number threshold (*P*_T_). (**c**) The breakdown of all permutations of *N* = 8 by the number of singularities. (**d**) Comparison of order recognition performances with the problem settings having the same singularity value. When the singularity number is small than 2, the near-field pattern outperforms other sources, whereas, with the largest singularity, the centre pattern exhibits the promptest responses.

For a given reward environment, consecutive 250-time slot machine plays were conducted. Such plays were repeated for *K*-types of different reward environments. The correct order recognition rate (COR) at the time step *t* was defined by the number of times when the order of the reward was correctly recognized at the time step *t* among *K*-types of different reward environments divided by *K*. The blue, green, and red curves in Fig. 7a represent the time evolution of COR. We observed that the near-field pattern most promptly achieved a higher COR than others. On the contrary, the centre pattern could not conduct correct order recognition for certain reward environments because COR does not converge to unity.

We then examined the order recognition ability as a function of the photon threshold *P*_T_. Figure 7b characterizes the number of steps when COR exceeds the value of 0.9 as a function of photon threshold *P*_T_. The near-field pattern, denoted by the blue curve, achieved smaller steps than others. The uniform and centre patterns, marked by the green and red curves, respectively, did not show evident *P*_T_ dependencies, which was understood from the fact that the uniform and centre patterns do not provide different spatial inhomogeneity by changing *P*_T_. The near-field pattern, marked by the blue curve, showed the minimum steps when *P*_T_ = 8. However, the difference in the time steps by changing *P*_T_ was not evident in this study, even in the case of a near-field pattern.

Finally, we examined order recognition from the viewpoint of singularities. There are a total of *K* = 40,320 assignments of the rewards to the eight slot machines. Each assignment can be represented by a Schubert matrix. Therefore, we can derive the singularities of the given assignment. Figure 7c shows the number of assignments, with the number being annotated with the bar graph, as a function of the number of singularities ranging from 0 to 6.

We evaluated the COR depending on the singularity number of the assignments, ranging from 0 to 5, as shown in Fig. 7d. When the singularity number was smaller than or equal to 2, the near-field pattern accomplished the fastest adaptation to COR = 1 or the perfect order recognition, whereas the COR by the centre pattern was limited below unity. Conversely, beyond the singularity number of 3, COR exhibited comparable performance irrelevant to the source-signal patterns. However, with the singularity number of 5, the centre pattern outperformed the others.

Interestingly, such a trend was contravariant to the trend of the frequency of the unique Schubert matrix analyzed in Fig. 6f. Therefore, the near-field pattern yields versatile Schubert matrices when there are few singularities and provides superior ordering performances for slot machine assignments with small singularities. With the centre pattern, on the other hand, the versatility of Schubert matrices is low when there are few singularities, and the order recognition ability is inferior when the singularity number of the problem is small. Overall, as shown in Fig. 7c, the dominant parts of all arrangements consisted of fewer singularity configurations; thus, on average, COR by near-field pattern predominantly exhibits higher values than the centre and uniform patterns, as summarized in Fig. 7a. Furthermore, the time step exceeding the COR of 0.9 by the near-field pattern was smaller than that in the uniform and centre patterns, as shown in Fig. 7b.

However, detailed insights into this phenomenon are warranted in future studies. For example, the time at which COR reached unity appears to be almost the same between the uniform and near-field patterns. Additionally, in the initial time, where the time was shorter than approximately 50, the centre pattern slightly outperformed the other two patterns.

Moreover, a deeper understanding of the underlying mechanism behind the relationship between the singularities of the source Schubert matrices and the order recognition is an exciting topic for future studies. For now, we believe that the abovementioned results demonstrate that the matching of the versatility of Schubert matrices with the number of singularities of the given problem plays a key role. Presumably, by subjecting a variety of different Schubert matrices matched with the singularity number of the given situation, the revision of the ordering proceeds efficiently. If a limited type of Schubert matrices is used, the order cannot be accurately recognized owing to insufficient comparisons. In a similar context, Okada et al., demonstrated that negative autocorrelation contained in a chaotic time series accelerates solving the two-armed bandit problems^33^, and this mechanism was recently evaluated based on correlated random walks^34^, which manifests the benefits gained by correlated randomness.

If one can know the singularity of the given problem as prior knowledge, the acceleration of the order recognition will be possible by engineering the source pattern that comprises appropriate singularities. For example, if the singularity number of the given problem is 5 or 6 only, the usage of the centre pattern is beneficial rather than the use of near-field or the uniform patterns. A clear understanding of related issues may be another interesting topic for future studies.

## Conclusion

We demonstrate an order recognition algorithm based on Schubert polynomials generated via irregular spatial distribution of photon transmission through a photochromic crystal photoisomerized by a local optical near-field excitation. The proposed strategy exploits the inversion relations and the singularities inherent in Schubert polynomials. A successful order recognition is demonstrated for 16 different reward probability slot machines. Furthermore, the performance of order recognition is quantitatively compared among the source Schubert matrices generated via near-field, spatially uniform, and strongly skewed patterns. We found that the singularity number contained in Schubert matrices and that of the given problems exhibit a clear correlation, suggesting that the order recognition will accelerate if the singularity number of the considered environment is presupposed. This study paves the way toward nanophotonic intelligent devices and systems by the interplay of complex natural processes and mathematical insights gained by Schubert calculus.

## Methods

### Definition of Schubert polynomials

The Schubert polynomial $\left\{ {{\varvec{S}}_{\omega } } \right\} = \left\{ {{\varvec{S}}_{\omega } (x)} \right\} = \left\{ {{\varvec{S}}_{\omega } (x_{1} , \ldots ,x_{N - 1} )} \right\}$^19–21,30^ in the symmetric group *S*_*N*_ is defined through the divided difference operator $\partial_{xy}$ given by

3 $$\partial_{xy} f = \frac{f(x,y) - f(y,x)}{{x - y}}.$$

Let the polynomial of the permutation with the maximum inversion number be given by

4 $${\varvec{S}}_{{\omega_{0} }} = x_{1}^{N - 1} x_{2}^{N - 2} \ldots x_{N - 1}^{{}} .$$

For a general permutation $\omega$, the polynomial is defined by

5 $${\varvec{S}}_{\omega } = \partial_{{\omega^{ - 1} \omega_{0} }} {\varvec{S}}_{{\omega_{0} }} .$$

In the case of the four-dimensional symmetric group $S_{4}$, the permutation with the largest inversion number is $\omega_{0} = [4321]$, which is represented by the anti-diagonal matrix shown in Fig. 4c. Its inversion number is 6, as shown in Fig. 4d. The corresponding Schubert polynomial is ${\varvec{S}}_{{\omega_{0} }} = {\varvec{S}}_{4321} = x_{1}^{3} x_{2}^{2} x_{3}^{1}$.

The Schubert polynomial of the permutation $[4231]$ is given by the divided difference operation on ${\varvec{S}}_{{\omega_{0} }}$ regarding *x*_2_ and *x*_3_, which corresponds to the interchange of the second and third rows in the Schubert matrix. Hence, ${{{\varvec{S}}_{4231} = \left( {x_{1}^{3} x_{2}^{2} x_{3}^{1} - x_{1}^{3} x_{3}^{2} x_{2}^{1} } \right)} \mathord{\left/ {\vphantom {{{\varvec{S}}_{4231} = \left( {x_{1}^{3} x_{2}^{2} x_{3}^{1} - x_{1}^{3} x_{3}^{2} x_{2}^{1} } \right)} {(x_{2} - x_{3} )}}} \right. \kern-\nulldelimiterspace} {(x_{2} - x_{3} )}} = x_{1}^{3} x_{2}^{{}} x_{3}^{{}}$ is obtained. Similarly, the Schubert polynomial of [2431] is given by the divided difference of ${\varvec{S}}_{4231}$ with respect to *x*_1_ and *x*_2_, which corresponds to the interchange of the first and second rows in the Schubert matrix. Therefore, ${{{\varvec{S}}_{2431} = \left( {x_{1}^{3} x_{2}^{{}} x_{3}^{{}} - x_{2}^{3} x_{1}^{{}} x_{3}^{{}} } \right)} \mathord{\left/ {\vphantom {{{\varvec{S}}_{2431} = \left( {x_{1}^{3} x_{2}^{{}} x_{3}^{{}} - x_{2}^{3} x_{1}^{{}} x_{3}^{{}} } \right)} {(x_{1} - x_{2} )}}} \right. \kern-\nulldelimiterspace} {(x_{1} - x_{2} )}} = x_{1}^{2} x_{2}^{{}} x_{3}^{{}} + x_{1}^{{}} x_{2}^{2} x_{3}^{{}}$ is obtained, which is the Schubert polynomial corresponding to the Schubert matrix shown in Fig. 4a. All Schubert polynomials, inversion numbers, and singularities of *S*_4_ are summarized in Table 1.

**Table 1:** Schubert polynomials of *S*_4_ and the corresponding inversion and singularity numbers.

Inversion number $l(\omega )$	Permutation $\omega$	Singularity number	Schubert polynomial ${\varvec{S}}_{\omega } = \partial_{{\omega^{ - 1} \omega_{0} }} {\varvec{S}}_{{\omega_{0} }}$
0	1234	0	1
1	2134	0	$x_{1}^{{}}$
	1324	1	$x_{1}^{{}} + x_{2}^{{}}$
	1243	1	$x_{1}^{{}} + x_{2}^{{}} + x_{3}^{{}}$
2	3124	0	$x_{1}^{2}$
	2314	0	$x_{1}^{{}} x_{2}^{{}}$
	2143	1	$x_{1}^{2} + x_{1}^{{}} x_{2}^{{}} + x_{1}^{{}} x_{3}^{{}}$
	1423	1	$x_{1}^{2} + x_{1}^{{}} x_{2}^{{}} + x_{2}^{2}$
	1342	1	$x_{1}^{{}} x_{2}^{{}} + x_{1}^{{}} x_{3}^{{}} + x_{2}^{{}} x_{3}^{{}}$
3	4123	0	$x_{1}^{3}$
	3214	0	$x_{1}^{2} x_{2}^{{}}$
	3142	1	$x_{1}^{2} x_{2}^{{}} + x_{1}^{2} x_{3}^{{}}$
	2413	1	$x_{1}^{2} x_{2}^{{}} + x_{1}^{{}} x_{2}^{2}$
	1432	1	$x_{1}^{2} x_{2}^{{}} + x_{1}^{2} x_{3}^{{}} + x_{1}^{{}} x_{2}^{2} + x_{1}^{{}} x_{2}^{{}} x_{3}^{{}} + x_{2}^{2} x_{3}^{{}}$
	2341	0	$x_{1}^{{}} x_{2}^{{}} x_{3}^{{}}$
4	4213	0	$x_{1}^{3} x_{2}^{{}}$
	4132	1	$x_{1}^{3} x_{2}^{{}} + x_{1}^{3} x_{3}^{{}}$
	3412	0	$x_{1}^{2} x_{2}^{2}$
	3241	0	$x_{1}^{2} x_{2}^{{}} x_{3}^{{}}$
	2431	1	$x_{1}^{2} x_{2}^{{}} x_{3}^{{}} + x_{1}^{{}} x_{2}^{2} x_{3}^{{}}$
5	4312	0	$x_{1}^{3} x_{2}^{2}$
	4231	0	$x_{1}^{3} x_{2}^{{}} x_{3}^{{}}$
	3421	0	$x_{1}^{2} x_{2}^{2} x_{3}^{{}}$
6	4321	0	$x_{1}^{3} x_{2}^{2} x_{3}^{{}}$

### Simulation details

In the performance comparison of the order recognition, we generated Schubert matrices from a spatially strongly skewed probability distribution called “centre”. Herein, we describe the details of this pattern. When the size of the matrix under study is *N* × *N*, the area of the centre is the set of elements (*i*, *j*) assuming the integer numbers of $\left\lceil {7N/16} \right\rceil \le i \le \left\lceil {9N/16} \right\rceil$ and $\left\lceil {7N/16} \right\rceil \le j \le \left\lceil {9N/16} \right\rceil$, where $\left\lceil * \right\rceil$ denotes the nearest integer greater than or equal to *. The detection probability of a pixel belonging to the centre was 100 times larger than that of the pixel outside the centre area.

We examined the statistics of the Schubert matrices regarding *N* = 8, 9, 10, 11, 12, 16, 20, and 24. The number of generated Schubert matrices for each case is summarized in Table 2. As the total number of different Schubert matrices is *N*!, the number of generated matrices should be sufficiently high to investigate the statistical characteristics. However, the computational demands are too high, especially when *N* is larger than 11 in the present computing environment. For these reasons, the number of the generated matrices was limited to 2 × 10^9^ when *N* is larger than or equal to 11.

**Table 2:** The number of generated matrices.

*N*	Number of generated matrices	*N*!
8	2 × 10^6^	4.03 × 10^4^
9	2 × 10^7^	3.63 × 10^5^
10	2 × 10^8^	3.63 × 10^6^
11	2 × 10^9^	3.99 × 10^7^
12	2 × 10^9^	4.79 × 10^8^
16	2 × 10^9^	2.09 × 10^13^
20	2 × 10^9^	2.43 × 10^18^
24	2 × 10^9^	6.20 × 10^23^

The computing environment was a workstation comprised of HPC Systems (CPU: Xeon Gold 6249R (20 cores, 3.1 GHz), RAM: 384 GB, OS: Ubuntu), and Matlab was the utilized software platform.

## Data Availability

The datasets used and/or analyzed in the current study are available from the corresponding author upon reasonable request.
